# Soil Odor as An Extra-Official Criterion for Qualifying Remediation Projects of Crude Oil-Contaminated Soil

**DOI:** 10.3390/ijerph17093213

**Published:** 2020-05-05

**Authors:** Saúl López-Aguilar, Randy H. Adams, Verónica Isidra Domínguez-Rodríguez, José A. Gaspar-Génico, Joel Zavala-Cruz, Edith Hernández-Natarén

**Affiliations:** 1 Facultad de Ingeniería, Universidad de Ciencias y Artes de Chiapas, Subsede Reforma, Carretera Reforma-Juárez Km. 6.5. Ra. Sta. Cruz, Reforma, Chiapas 29500, Mexico; sloaguilar@hotmail.com; 2 División Académica de Ciencias Biológicas, Laboratorio de Remediación, Universidad Juárez Autónoma de Tabasco, Carretera Villahermosa-Cárdenas km. 0.5 s/n, Villahermosa, Tabasco 86150, Mexico; tazvro@hotmail.com (V.I.D.-R.); genico220@gmail.com (J.A.G.-G.); 3 Colegio de Postgraduados, Campus Tabasco. Periférico Carlos A. Molina s/n, H. Cárdenas, Tabasco 86500, Mexico; zavala_cruz@colpos.mx (J.Z.-C.); nataren.edith@colpos.mx (E.H.-N.)

**Keywords:** perception, odor, bioremediation, natural attenuation, petroleum

## Abstract

Unfortunately, many property owners in southeastern Mexico do not trust environmental authorities, and the de facto method they use to evaluate the progress in environmental remediation projects is soil smell. This criterion was evaluated to determine if it was reliable to assess soil fertility and toxicity. Three soils (Fluvisol, Gleysol, and Arenosol), were contaminated with 2% medium or heavy crude oil (30.2, 17.1°API, respectively), and treated for 18 months to simulate bioremediation or natural attenuation. Every two months, field capacity, water repellency, hydrocarbon concentration, acute toxicity and soil odor were measured. Odor was measured in controlled conditions with a group of unexperienced panelists. During remediation, the Fluvisol and Gleysol were perceived to have an odor intensity between slight to low, and were considered acceptable. Meanwhile, in the Arenosol, the odor intensity was between low to medium and was considered unacceptable. After treatment, the hydrocarbon concentration was reduced to low levels, very near Mexican norm, and all the soils, including the Arenosol, were perceived to have an intensity between neutral to slightly agreeable, were considered acceptable, and no toxicity was observed in the earthworm bioassay (no false positives). However, in various soil samples from the Fluvisol and Arenosol, important risks were present with respect to field capacity and water repellency. Due to these observations, even though soil smell may be a trustworthy guide to soil toxicity, it does not ensure that the remediated soil’s fertility has been restored.

## 1. Introduction

In Mexico, the regulations for the characterization and remediation of hydrocarbon-contaminated sites are governed by a system of laws, regulations and norms, which together try to guarantee that site remediation achieves three objectives: 1) protection of the environment, including living beings and biological processes, 2) protection of public health, and 3) restoration of the site such that it can be used according to its natural vocation or any activity considered in a program of urban development (Ley General de Equilibrio Ecológico y Protección al Ambiente—LGEEEPA, Art.134, fracc. V, and Art. 136) [1]. Among those actions which should be considered in the remediation of contaminated sites are the reduction in toxicity, the potential for leachates (and contamination of water bodies and aquifers), as well as those measures that permit the re-establishment of the physical and chemical properties associated with soil fertility [2]. 

To try to assure compliance with the Maximum Permisible Limits (MPLs) in the Mexican Norm, a process has been established to accredit individuals, companies and institutions to be legally reconized for sample collection and analysis of soil from contaminated/remediated sites. However, this supposes that the social mechanisms exist to assure that the application of the MPLs are indeed being met [3,4]. Nonetheless, the individuals and institutions that watch over the remediation companies can be corrupted. There is a possibility that the persons responsible for certain areas of accredited laboratories would be willing to report results in favor of the remediation company in exchange for an extra-official gratuity. This situation can also present itself with respect to some persons within the environmental authorities charged with watching over the remediation companies, as well as with the project supervisors responsible for the remediation project on the part of petroleum companies. This possibility of personal corruptiblity puts the true compliance of the MPLs in question. Furthermore, there is no guarantee that remediated soils that achieve the MPLs will indeed have their fertility restored [5,6]. Often, these soils may still suffer from water repellency and reduction in the field capacity. In the case of some soils, especially red clay petroleum-contaminated soils, compaction may also be a problem. According to the experiences of of various persons working in remediation companies, the de facto criteria used by property owners, remediation personnel, and site supervisors are organoleptic, especially soil odor [7,8].

The sense of smell, as well as taste, is a chemical sense, due to its capacity to detect volatile chemical substances in the environment at the moment that we breathe. Compared to any other of our senses, the sense of smell is 10,000 times more sensitive, and the recognition of smells can be immediate, even if the compound perceived is found in low concentrations in the air. This information is sent straight to the brain [9,10,11]. Even though the sense of smell is important, it is one of the senses least studied due to its subjective nature, and measuring smell has required the empirical development of sensory techniques [12]. Specifically, there is no literature or norm that refers to the evaluation of odor perception of crude-oil contaminated soils that could be considered as a criterion for evaluating the effectiveness of remediation projects. Those studies that do exist on odor are scarce and are focused principally on the food, perfume, and wine industries. However, this is starting to change. Smell is being studied more, specifically with respect to the acceptability of unpleasant odors from production plants and their relationship to the perceived public good. The existent investigations allude to the impacts from unpleasant and offensive odor emissions to community residents. This has been with the purpose of comparing intensity and the acceptation level [13,14], general contamination from odors [15], odors coming from wastewater treatment plants [16], sanitary landfills [17,18], agriculture and livestock raising [19,20,21], as well as paper processing plants, refineries, fertilizer plants, and thermal-electric plants. From these studies, one can conclude that most countries do not have adequate emission control legislation to protect community members from extremely unpleasant odors, presenting areas of opportunity for resolving one of the main sources of social anger directed at economically productive activities [12,22].

The hypothesis of the present study was that the perception of hydrocarbons in remediated soils can be used as a trustworthy criterion to assure that the toxicity is in the same range as background, and that soil fertility is restored. The objective of this research was to evaluate smell as a criterion that can discriminate the effectiveness of the remediation of soils contaminated with medium and heavy crude oil.

## 2. Materials and Methods 

### 2.1. Selection of Soil and Crude Oil Samples, Experimental Cell Preparation, Simulation of Contamination and Remediation

To simulate a terrestrial oil spill and subsequent bioremediation or natural attenuation, soils were selected and located based on the soil classification proposed by Palma-López [23] for Tabasco State. Three soils with different textures representative of the region were selected: an Arenosol, a Gleysol and a Fluvisol, which were identified by their characteristics in the field and in soil profiles according to Zavala and García [24]. These soils are classified in Mexico according to the WRB system [25]. Since many countries also use the Soil Taxonomy (ST) system [26,27], the approximate equivalents are also given in ST: Arenosol ≈ Psamment, Gleysol ≈ Aquent, and Fluvisol ≈ Fluvent. They correspond to a sandy coastal soil in vegetated dunes (Haplic Arenosol), a clayey, floodable soil from a marshy area (Eutric Gleysol), and a medium textured river levee soil (Eutric Fluvisol). The physical-chemical properties of the uncontaminated soils, as well as their coordinates, are shown in Table S1.

Approximately 600 kg was collected for each soil at a depth of 0–30 cm. This was done considering that for the majority of crops, most of the roots are at this depth [28]. Straight shovels were used to hand dig and to collect representative material from the 0–30 cm depth. The soil collected was placed in plastic-fiber gunny sacks and transported to the laboratory. The Arenosol was collected in a coastal area previously described by Adams [29]. The vegetation at this site was principally humidicola grass (*Brachiaria humidicola*), and coconuts (*Cocos nucifera*). The Gleysol was located in a floodplain near the Blasillo River (881 m from the river) and has been described previously [24,30]. The vegetation at this site consisted predominantly of induced, flood-tolerant pastures. Likewise, the Fluvisol was obtained from a property near the Blasillo River (146 m from the river) and has been described previously by Morales Bautista [30]. The soil samples were open-air dried and plant roots removed. Later, the soils were ground, screened and homogenized, and then characterized for physical and chemical parameters before being contaminated. The methods used for these determinations are presented in Section 2.2.

The collection of crude oils was carried out in different oil wells in the Samaria Production Unit of the state run oil company, Petróleos Mexicanos. The verification of °API was carried out using the ASTM D6822-12b method [31] using Kessler ASTM 52HH y 53HH thermohydrometers with different °API scales, and a thermometer as per Guzmán-Osorio et al. [32]. The medium crude oil sample (30.23 °API) was obtained from well No. 7105 and the heavy crude oil sample (17.7 ° API) was obtained from well No. 851, both in the Samaria Oil Field, in Tabasco State (Mexico).

**Figure 1:**
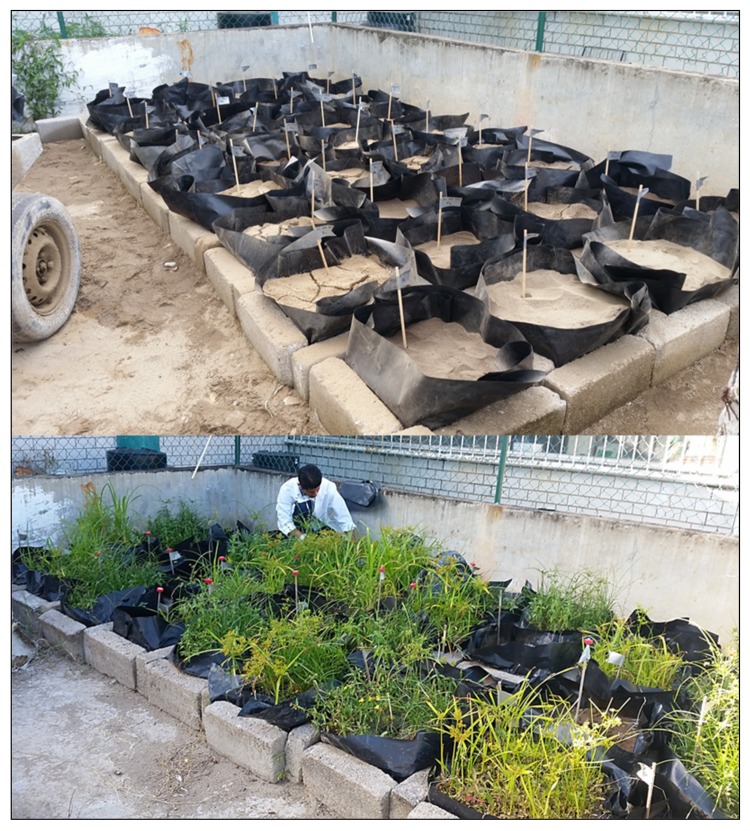
Bioremediation and natural attenuation treatment cells. Above: recently prepared treatment cells. Below: cells after several months of treatment. Note that many cells became naturally vegetated with weedy plants, principally grasses and sedges.

An outdoor patio with a concrete floor was used to build the treatment cells (36 total). The cells were built using cement blocks on top a ~3 cm layer of sand for drainage. The cell dimensions were 40 cm × 40 cm by 20 cm deep. In each cell, on top of the sand layer, a ~1 mm high density polyethylene plastic sheet was placed, with holes punched in the bottom to help drainage. The capacity of each cell was 40 kg, as per Adams et al. [2], (see Figure 1). Afterwards, soil was contaminated at a concentration of 20,000 mg/kg of medium crude oil (~30 °API) or heavy crude oil (~18 °API) as per Marín-García et al. [33], using a small cement mixer with a 255 L capacity, mixing at 15 RPM. The contaminated soils were placed in the treatment cells on top of the plastic sheet, and their orientation in the patio was randomized. Newly contaminated soil from the treatment cells were characterized for field capacity, water repellency, and hydrocarbon concentration (Total Petroleum Hydrocarbons—TPH).

Semi-passive bioremediation was simulated by adding an inorganic fertilizer, N-P-K 17:17:17, also known as “Triple 17” (Fertilizantes GL, S.A. de C.V.), to a final concentration of 300 mg N kg^−1^ of soil, and well mixed. The treatment cells were left outdoors for 18 months, during which they received rain water, solar radiation, growth of (uncultivated) vegetation, and other climatic variables that were not directly controlled (Figure S1). This experiment was carried out in a tropical monsoon climate (Am in the Köppen classification system) with an average temperature of ~28 °C, annual precipitation of ~2100 mm, and relative humidity usually between 50–85%, as described by Adams et al. [34]. During this period the petroleum gradually biodegraded simulating a bioremediation project. Alternatively, no nutrients were added during this period, to simulate natural attenuation by microbial activity, volatilization, dilution, adsorption, complexing, and abiotic transformation. This is a simple, low-cost technique [35].

### 2.2. Initial Characterization of Soil and Determination of Fertility Parameters

The initial characterization of the soils included electrical conductivity, bulk density, pH, nitrogen, phosphorous, potassium, % organic material, and texture, which were determined according to Mexican Norm NOM-021-SEMARNAT-2000 [36], solid density which was determined by the method proposed by Domínguez and Aguilera [37]; and field capacity which was analyzed according to the Colman column method [38,39] as per Zavala et al. [40]. (Table S1). 

One of the major impacts to soil fertility in petroleum-contaminated soils, is interference with the normal soil–water–plant relationship. This is due to the formation of thin laminates of hydrocarbons on what would otherwise be wettable surfaces of soil aggregates [29,41]. This appears to be the cause of soil water repellency [30], as well as a reduced moisture content at 100% field capacity. For this reason, water repellency and field capacity were evaluated as the main impacts to fertility in the petroleum-contaminated (and remediated) soils. Field capacity was evaluated as mentioned previously. Soil water repellency was run in two ways: 1) for severity (Molarity Ethanol Drop—MED) by the method proposed by King (1981) with modification by Adams [29] et al. (2008a); and 2) for persistence (Water Drop Penetration Time—WDPT), also as per Adams [29] (Tables S2, S3 and S4).

For the present study, compaction was not evaluated, being more prevalent in petroleum-contaminated soils with predominantly kaolinte clays [6], or in soils in which surface and subsurface horizons have been mixed during the remediation process [5]. As this study only involves surface soil, with very little clay (Arenosol) or with predominantly smectite clays (Fluvisol, Gleysol), compaction was not considered a primary fertility parameter likely to be impacted by crude oil contamination.

### 2.3. Critical Moisture Content

Generally, water repellency is determined in dry soil. However, in the field it has been demonstrated that repellency depends on the moisture content of the soil. Even during the driest part of the year there is still a little moisture in the soil that could mitigate water repellency [42,43]. For this reason, Critical Moisture levels were determined. This was done by measuring the penetration times of water drops at different soil moisture contents according to Guzman-Osorio and Adams [44].

### 2.4. Total Petroleum Hydrocarbons

Total Petroleum Hydrocarbons (TPHs) were quantified by the EPA method 418.1 [45] according to Adams et al [2], Mayo-López et al. [46] and Adams and Guzmán-Osorio [47] using perchloroethylene as a solvent, and the medium and heavy crude oil to prepare independent calibration curves, as per Adams [29]. For both the medium crude and heavy crude, there were slightly asymptotic functions of concentration vs. absorbance. The linear form of this function for medium crude showed a linearity of R = 0.96, with a Limit of Quantitation of 2349 mg/Kg, a Limit of Detection of 443 mg/Kg and a Sensibility of 263 mg/Kg. For the heavy crude, the linearity was R = 0.97, with a Limit of Quantitation of 2096 mg/Kg, a Limit of Detection of 388 mg/Kg and a sensibility of 257 mg/Kg. This parameter was determined at the beginning of the remediation treatment process and every two months thereafter up until 18 months.

### 2.5. Earthworm Bioassays

These tests were run with *Eisenia foetida* using a modification of Protocol 207 of the OECD (1984) according to Domínguez-Rodríguez et al. [48,49]. The bioassays were run by direct contact with the soil, without dilution. The tests were done after the fourteenth and sixteenth month, and at the end of the treatments. The direct contact test consists of exposing earthworms to a thin layer of moist soil, using 10 replicates per test. The test jars were covered with gauze to assure air availability and to avoid escape. The tests were monitored for 48 hours. Those worms that survived this period were washed with distilled water, dried, and weighed to determine biomass.

### 2.6. Odor Perception Test

To carry out the odor perception tests, a temporary installation was employed as per ISO 8589:2010/A1:2014 [50,51]. A semi-quantitative questionnaire was used that was composed of three yes/no or multiple choice questions to evaluate the intensity and acceptability of the samples. The multiple choice questions were based on an odor scale of seven levels from “without odor” to “very strong odor” for intensity, and “very pleasant” to “very unpleasant”, for acceptability (Figure S2). These results were analyzed using descriptive statistics including percentages, averages and standard deviation. For those data obtained on the tests of intensity and acceptability (with ordinary variables), and their relationship to parameters such as field capacity, water repellency, TPH concentration and toxicity (ratio variables), a non-parametric test of bi-variable regression was used, Spearman’s Rho, with a level of confidence of α = 0.05. For this, the IMB SPSS Statics 20 package (International Business Machines Corp., Armonk, NY, USA) for Windows was used [52,53].

Panelist received information previous to the test with respect to test objectives and procedure. For the first two test on odor, 16 and 11 male panelist (judges) between 30 and 50 years old participated, from the university maintenance department. For the following tests, 15 panelists participated, male students between 19 and 25 years old from the environmental engineering bachelor´s program at the university. Samples were previously prepared in uniform jars covered with brown paper. Humidity in the samples was homogenized at 70% of field capacity, and the room conditions were ~24 °C, and ~50% relative humidity. To each panelist, 36 jars were assigned, only identified by a code unknown to the panelists. The order for testing the samples was randomized. Each panelist gently unscrewed one jar at a time, counted two seconds, brought the jar to within five centimeters of their nose, and softly inhaled to perceive the sample odor, usually for five to ten seconds. 

The samples were made up of three kinds of soils contaminated with two kinds of crude oil and subjected to simulations of either semi-passive bioremediation or natural attenuation. Panelists were offered room-temperature drinking water and slices of apple to clear the palate during programmed pauses. After each set of nine samples, the panelists had a brief, five minute break if they felt they needed it. Whenever the samples tested did not have strong odors, the time between testing each sample was relatively short, without the necessity of limiting the number of samples in each session [52,53].

### 2.7. Ethics 

The research protocol was evaluated and approved by the Divisional Research Committee of the Biological Sciences Academic Division of the Univerisidad Juárez Autónoma de Tabasco (Folio No. 395-2017) after considering protection to human health, dignity and scientific rigor. More details can be found in Supplementary Materials, including the consent form (Figure S3) and the panelist’ data form (Figure S4). 

## 3. Results

### 3.1. Characterization of Initial and Treated Soil Samples

The detailed results of initial soil characterization and major changes in TPH resulting from the semi-passive bioremediation or natural attenuation are shown in the Supplementary Material accompanying this article (Table S1, Figures S5–S8). In general, from an initial concentration of 20,000 mg/Kg, the TPH were reduced roughly 80% or more, into the range of about 2200 to 4300 mg/Kg, and very near the Maximum Permissible Limit (MPL) in Mexican norm (3000 mg/Kg) [54]. However, even at these very low levels, the ability of the soil to maintain moisture (field capacity) was much below uncontaminated levels, to about 50–70% in the Fluvisol (river levee soil), to about 45–60% in the Gleysol (clayey, floodable soil), and to about 40–75% in the Arenosol (sandy coastal soil).

### 3.2. Water Repellency

With respect to soil water repellency, the results were mixed. Initially, prior to contamination with crude oil, all the soils were wettable (not water repellent). However, after contamination, and even after 18 months of treatment in a humid, tropical environment, many samples were still water repellent. This was most apparent in the sandy soil (Arenosol). In this soil, all samples showed a severity classified as “very severe” (King, 1981) [55] and a persistence classified as “extreme” [42]. In the river levee soil (Fluvisol), the severity was moderate to severe in the samples contaminated with medium crude, and very severe in the samples contaminated with heavy crude. Likewise, with respect to persistence, the final Fluvisol samples showed strong to extreme values. In contrast, in the clayey, floodable soil (Gleysol), in those samples contaminated with medium crude, after 18 months of treatment, the severity was null and the persistence was only slight. However, in the Gleysol samples contaminated with heavy crude, the final values for severity were low to moderate, and had a persistence classification as “strong”.

These evaluations of water repellency are conventionally carried out on dry samples in standardized tests. However, it has been shown that even during the dry season, the soil usually has at least a little moisture, and that this residual moisture may partially mitigate the water repellency [29,33,56]. For this reason, some of the water repellent samples were selected for further study—to determine the critical moisture content at which water repellency may manifest. This is especially important for those samples with more moderate water repellency. Final samples for the Fluvisol contaminated with medium crude were selected (with and without fertilization) as well as the sample contaminated with heavy crude and without fertilization (for comparison). Likewise, the Gleysol samples contaminated with heavy crude were also selected. The Arenosol samples had such high water repellency, and the Gleysol samples contaminated with medium crude had such low water repellency, that they were not included in the evaluation (Table S5).

### 3.3. Critical Moisture Content

In the Fluvisol samples contaminated with medium crude, the critical moisture content to manifest slight water repellency (for a drop a water to absorb in 60 seconds or less) was about 9–11.5% H, and to be completely wettable (for a drop a water to absorb in five seconds or less) was about 14–15%. Likewise, in the Fluvisol contaminated with heavy crude and with fertilization, these values were about 13.5% H and 17% H, respectively. Thus, for these soils, in natural, field conditions, the moisture content would have to drop to about 14–17% to just start to manifest water repellent conditions, and even at moisture contents in the range of about 9–14% H, the water repellency would only be slight (Table 1). In a nearby similar soil (only about 60 m from the Fluvisol in question) Marin-García [33] found that even during the dry season, the in situ moisture content of the soil was 14.8% H at the surface and 15.5% H in soil crevasses. These values are generally higher than those found for the critical moisture content in the Fluvisol in the present study, and therefore, it is unlikely that in the field, water repellency would manifest in these samples, or if so, only slightly and for a brief period of the year.

In relation to the Gleysol samples studied, the moisture content to be completely wettable were about 17–18% H and to be only slightly water repellent were about 14–14.5% H. Adams [29] found that in these kinds of soil, even during the driest part of the year the soil maintained 80% of field capacity. In this soil that would be almost 30% H, much higher than the critical levels. As such, it is very, very improbable that in this soil water repellency would present itself, even during the dry season.

### 3.4. Field Capacity

To sum up the results on the soil–water relationship in the contaminated and treated samples, even after treatment, all samples had less field capacity, in the range of about 40–75% of initial levels (Figure S9). Additionally, the anticipated water repellency in the field was very high for the sandy soil, generally high for the river levee soil, especially if contaminated with heavy crude and without added nutrients, but moderate to null in the other river levee soils studied, and practically null in the clayey floodable soil. Considering both of these parameters, we can conclude that the sandy soil was very affected, the river levee soil moderately affected (but still with some potential problems) and the clayey, floodable soil, basically not affected, principally due to it low-lying aspect in the landscape and very humid to flooded conditions year-round [57].

### 3.5. Toxicity

In these tests, no mortality was observed and very little signs of stress, with only a slight loss of biomass (overall weight). Thus, even though the hydrocarbon concentration was not reduced to really low levels, the acute toxicity due to hydrocarbons was essentially eliminated (Table S6). However, this is not to say that the conditions would be adequate and favorable for the biota in general on a longer time scale, especially considering the findings of the field capacity reduction and water repellency. It is possible that the soil would not be toxic, but could not retain sufficient moisture to maintain some of the more sensitive soil fauna, especially earthworms, nematodes, and the fine roots of some plants.

### 3.6. Soil Odor Perception

In practice, personnel in remediation companies, oil companies, government authorities and affected land owners often use soil smell to gauge the degree of hydrocarbon contamination in soil and remediation effectiveness. This is often done as a field test in lieu of more costly and longer chemical analysis, even though in Mexico, as in almost all other countries, there is no legal framework to evaluate the results of the smell test. This is for several reasons, among which it is generally considered to be difficult to measure something as subjective as smell [58]. In addition to smell, there are a few other extra-official criteria that are used to evaluate soil contamination/remediation, such as the absence of an oily sheen in puddles when it rains, and the growth of vegetation (especially pastures) post-remediation.

For the soil odor perception tests, there was one question on intensity (“Does it smell like crude oil?”) that was graded according to a scale, ranging from “no odor” (level 1), to “very strong odor” (level 7). Likewise, the panelists were asked to respond to an acceptability question (“It is pleasant, or unpleasant?”) according to a scale from “very pleasant” (level 1) to “very unpleasant” (level 7), (Table 2). According to this scale, an intensity of 3 or less was generally considered to be satisfactory and a level of acceptance of 4 or less (neither pleasant nor unpleasant—neutral) was considered to be acceptable. In addition, a question was presented with respect to if the panelist considered the soil to be apt for planting vegetation (yes/no).

**Figure 2:**
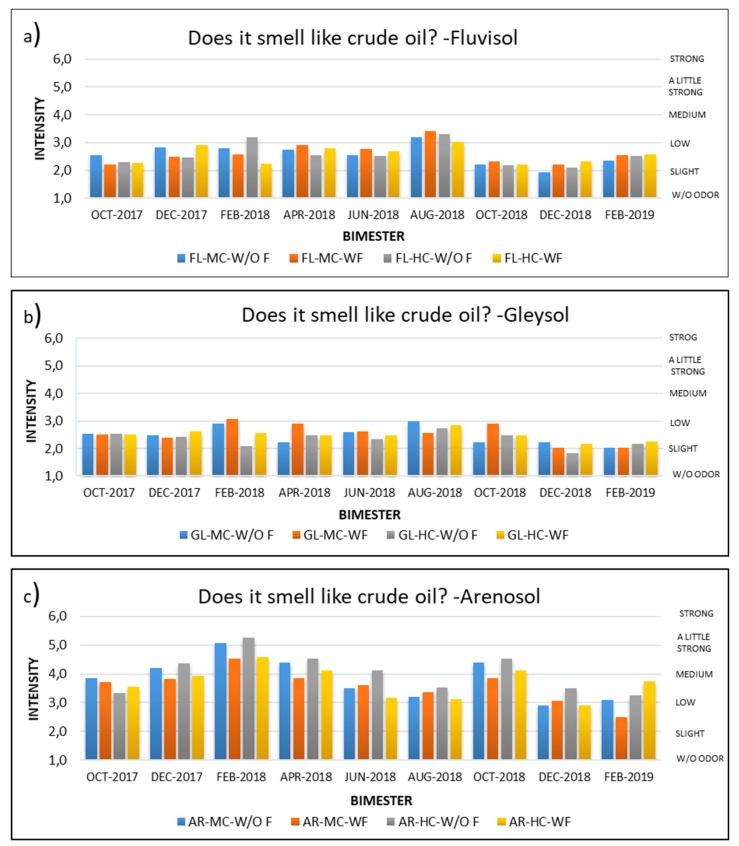
Intensity of petroleum odor in soil. **a**) In Fluvisol, **b**) in Gleysol, **c**) in Arenosol. Values are averages of three replicates. FL = Fluvisol; GL = Gleysol; AR = Arenosol; MC = Medium Crude Oil; HC = Heavy Crude Oil; W/O F = Without Fertilizer; WF = With Fertilizer.

Nine odor perception tests were conducted during the 18 month study. The results of the intensity values are shown in Figure 2. In the Fluvisol and Gleysol samples, the average intensity was almost always “low” or less, and varied slightly during the treatment, generally being a little lower in Gleysol than in Fluvisol. The final values for the Gleysol samples were slightly less than those reported during the test. For both soils, the final intensity values were in the “slight” to “low” range (values of 1–2). However, in the Arenosol samples, the intensity values were more variable, generally ranging from “slight” to “a little strong” and with a tendency to reduce the intensity in the later bimesters of the treatments. These differences between Gleysol, Fluvisol and Arenosol may have to do with the amount of and type of fine particles in the soils. In general, the Gleysols in this region have a high quantity of fine particles (approx. 60–70%), mostly made of smectite clays, while Fluvisols have less, and Arenosols generally have less than 3% fine particles [23]. These fine clays have a large amount of surface area and are able to adsorb contaminants, such as hydrocarbons [33], thereby reducing their bioavailability, leachate potential, and consequently, smell. Thus, the soil with the greatest amount of fine clays has the lowest hydrocarbon smell and the soil with the lowest amount of fine clays has the highest hydrocarbon smell.

**Figure 3:**
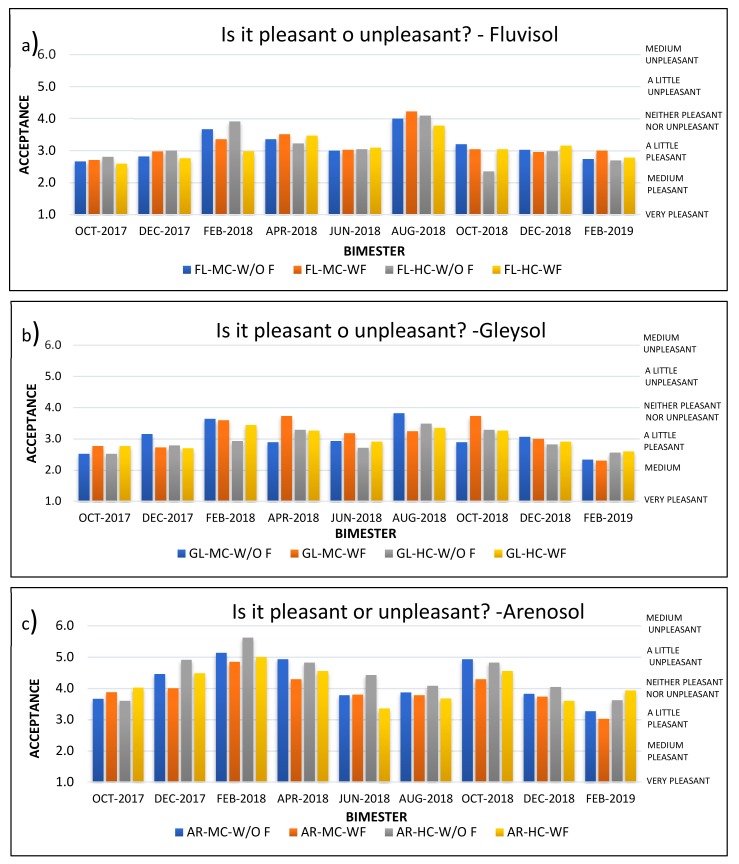
Acceptance level for odor in soil. **a**) In Fluvisol, **b**) in Gleysol, **c**) in Arenosol. Values are averages of three replicates. FL = Fluvisol; GL = Gleysol; AR = Arenosol; MC = Medium Crude Oil; HC = Heavy Crude Oil; W/O F = Without Fertilizer; WF = With Fertilizer.

Likewise, with respect to acceptability (Figure 3), the Fluvisol and Gleysol samples generally had low values, for the most part less than neutral (value of 4), with the average values in the Fluvisol usually being a little higher than in the Gleysol. The final acceptability values for these soils were in the “slightly pleasant” to “medium pleasant” range, all considered to be acceptable. Meanwhile, the acceptability in the Arenosol samples was more variable and higher, for the most part in the “slightly pleasant” to “slightly unpleasant” range. Only in the last bimester did the average values consistently fall at or below the acceptable range, “neither pleasant nor unpleasant”. These tendencies (low values for Fluvisol and Gleysol, with Fluvisol being a little higher, and much higher values for the Arenosol), are consistent with those observed for intensity and probably related to the same mechanism previously explained. It is noteworthy that although the Arenosol generally had higher values (lower acceptance), even this soil was considered acceptable at the end of the treatment. It should be mentioned though, that these are all average values, and individual values may change according to personal criteria and ability to perceive odors.

Crude oil tends to have a peculiar odor that depends on the composition of hydrocarbons in the oil, as well as the amount of sulfur containing compounds [59]. These kinds of odor can be detected by human smell, even in low concentrations in the air [10,11]. With respect to odors, the Mexican environmental legislation only makes reference to contamination by bad odors, without giving more relevance to this issue, and certainly not quantifying the odor.

The General Law on Ecological Equilibrium and Protection of the Environment [1] establishes in Article 5 that the federation has competence (legal authority) to regulate on the prevention of contamination of odors. Furthermore, Article 7 establishes the states as responsible for the prevention and control of generalized contamination caused by the emission of odors that are noxious and generated by fixed sources (such as industrial installations) as well mobile sources, and that are not of federal competence.

### 3.7. Relationship between Smell, Soil Fertility and Toxicity

By use of inferential statistics, the possibility that there exists some relationship between smell intensity/acceptance (as dependent-ordinal variables) and the fertility parameters such as hydrocarbon concentration, field capacity, and water repellency (as independent-ratio variables) was analyzed. Given the data’s non-parametric characteristic, the statistical method via Spearman’s rho was employed, using the IBM software SPSS Statistics 20 [60]. According to the results of this analysis, it was determined that the relationships between smell and fertility factors were minimal. The values for Spearman’s rho were highly irregular, generally low and in some cases, inverse of what might be expected. (Table 3). For example, with respect to the hydrocarbon concentration vs. smell, the Spearman’s rho values for TPH vs. smell intensity ranged from 0.217 to 0.619 in all of the soils, and for TPH vs. smell acceptance the values ranged from −0.167 to 0.577. For field capacity vs. smell intensity, Spearman’s rho values ranged from −0.424 to 0.617, and compared to smell acceptance the values ranged from −0.489 to 0.594. Likewise, for water repellency (severity), the values for Spearman’s rho ranged from −0.043 to 0.800 for smell intensity and from −0.202 to 0.544 for smell acceptance. As seen from these low and even inverse values, smell is not strongly related to these fertility parameters in soil in any reliable way.

As previously stated, with respect to acute toxicity by direct soil contact, no mortality nor high stress factors (such as expulsion of coelomic/bloody fluid, inflammation of the clitellus) were observed, only slight reduction in biomass, which was similar in the three soils studied. It is worth mentioning that during this direct contact bioassay, it is important to control humidity so that the test organism does not dehydrate during the test period. It appears that the level of degradation that occurred in the treatments after 18 months was able to reduce the toxicity of the hydrocarbons in the soil. This was congruent with the perception of smell in terms of intensity and acceptance. Thus, when the perception of soil odor was considered acceptable (level 4, neither pleasant nor unpleasant—neutral), the soil really did appear to be free of acute toxicity. This is reasonable, considering that for most mammals, including humans, taste and smell are used to identify and avoid hazardous substances, such as naturally occurring hydrogen sulfide from sulfur springs, some poisonous plants, and potentially pathogen-containing materials, like decomposed food stuffs and feces. This is also reasonable considering that crude petroleum is a naturally occurring substance and that it is found at the earth’s surface in petroleum seeps (tar pits).

In Table 4, Table 5 and Table 6, a summary of data is presented relevant to the ability to use smell to determine if a petroleum-contaminated soil has been adequately remediated. This is based on the research question: when they say “it is good to plant”, is it really satisfactory? For this purpose, final values (after treatment) were included on water repellency (severity and persistence), field capacity, and toxicity and compared to the odor intensity and acceptance.

For the river levee soil (Fluvisol), the fertility parameters evaluated in the laboratory indicate that even after treatment, there may be problems with the soil–water relationship. This soil still presented water repellence severity in the range of moderate to very severe and a persistence from strong to extreme. As mentioned previously, is it probable that under field conditions, the in situ moisture content never drops below the critical moisture content. Thus, in the field, it is improbable that this soil would manifest soil water repellency [29,33]. Nonetheless, there was still the issue of a reduced field capacity. Even after treatment, the field capacity was still 28.3% to 43.5% less than in uncontaminated soil. Accordingly, it is unlikely that the soil recovered its fertility completely. Consequently, with respect to fertility, smell is not reliable (presents false positives).

In contrast to fertility, in the Fluvisol, the treatments (either by passive bioremediation of natural attenuation) did result in the reduction of acute toxicity to levels in which there was no mortality nor signs of stress to the test organism. In these (final) samples, both the levels of smell intensity and acceptance were satisfactory. Thus, the research question: when they say “it is good to plant”, is it really satisfactory, at least with respect to toxicity, is affirmative. No false positives were found (with acceptable smell but toxic conditions). Thus, it should be safe to let animals graze, but due to poor field capacity, there may not be enough moisture to maintain the pasture. If post-remediation, some steps were made to increase field capacity (by addition of organic amendments, for example), it is possible that the soil could recover. If under these conditions, in addition to smell, tests were also run to assess a recovered field capacity, this might be sufficient to confirm site remediation and adequate restoration.

The results in the clayey floodable soil (Gleysol) were similar to the Fluvisol with respect to toxicity—no false positive were found (Table 5). With respect to the soil–water relationship, however, the situation is more complex. As mentioned previously, due to the low-lying nature of this soil, it is flooded or humid year round, and the water repellency data generated in the laboratory are probably not relevant to the conditions in the field. It is very unlikely that this soil would suffer from insufficient moisture, even during the driest part of the year. Consequently, the fertility question, with respect to water repellency or field capacity, becomes moot. Only the toxicity question remains, and in this case, as in the Fluvisol, there were no false positives. Thus, it appears that for Gleysols, at least those with similar conditions to this one (clayey, alluvial, smectite-rich soil, in a tropical-monsoon climate), smell may be a reliable criteria for judging the acceptability of a remediation project. When the smell is acceptable, there is no acute toxicity, and allowing cattle to graze should be all right.

Among the three soils studied, the sandy soil (Arenosol) was the most divergent. With a very low quantity of fine particles, it is likely to present greater bioavailability of the contaminants, and consequently, greater smell intensity, and less acceptability. During most of the treatment period the smell acceptability was unsatisfactory. However, in the final bimester, the acceptability was reliably satisfactory. This was consistent with the toxicity data (no false positives). Thus, when the smell was acceptable, the toxicity was also null. However, with respect to fertility, the situation was even more extreme than in the Fluvisol. The final samples of the Arenosol all had a reduced field capacity from 20 to 59% less, the water repellency severity was classified as “very severe” and the persistence was classified as “extreme”. Thus, even though the smell was acceptable, the water repellency and field capacity were affected—all false positives. In this soil more than any other, smell was inadequate to evaluate the effectiveness of remediation. Even if the soil was not toxic, the soil–water relationship was very heavily affected.

## 4. Conclusions

In this study, the simulation of bioremediation and natural attenuation resulted in the reduction in the hydrocarbon concentration in the soil to levels very near the Maximum Permissible Limit according to Mexican environmental norm. However, not all soils recovered their fertility, especially with respect to field capacity in those soils with medium to coarse textures. Nonetheless, upon finalizing the treatment period, acute toxicity was not encountered in any of the soils. With respect to the perception of soil odor, the intensity for the Fluvisol and Gleysol were generally perceived to be between slight to low, but in the sandy soil (Arenosol) it was generally between low to medium. Regarding smell acceptance, the Fluvisol and Gleysol had generally pleasant levels even during the remediation, while the Arenosol generally had unsatisfactory levels during the remediation. However, upon finishing the remediation treatment period, all the soils studied had satisfactory levels of smell intensity and acceptance. Considering these results, soil smell can be considered to be a reliable criterion for evaluating acute toxicity in these soils, and probably reliable for evaluating fertility in the Gleysol. However, in the Fluvisol and Arenosol, this criteria by itself would not be reliable for evaluation of soil fertility and should be avoided.

## Figures and Tables

**Table 1:** Critical humidity in Fluvisol and Gleysol.

Sample	HTP (mg kg^−1^)	MED 10 (Molarity)	H.C. 5 s (%)	H.C. 60 s (%)	Observations
FLMC-W/O F	2996.91	2.11	13.89	8.94	Probably not repellent in field conditions
FLMC-WF	3855.72	2.81	14.99	11.49	
FLHC-W/O F	4335.04	3.46	17.08	13.61	Probably not repellent in field conditions
GLHC-W/O F	3753.90	0.62	17.08	14.09	Probably not repellent in field conditions
GLHC-WF	4033.87	2.00	18.04	14.45	

FL = Fluvisol; GL = Gleysol; MC = Medium Crude Oil; HC = Heavy Crude Oil; W/O F = Without Fertilizer; WF = With Fertilizer; H.C.5 = Critical moisture (WDTP = 5 s); H.C.60 = Critical moisture (WDTP = 60 s).

**Table 2:** Criteria and values for odor perception.

Odor Intensity Does It Smell Like Crude Oil?		Acceptance Level for Odor Is It Pleasant or Unpleasant?	
Criterion	Value	Criterion	Value
Without odor	1	Very pleasant	1
Slight odor (barely perceptible)	2	Medium pleasant	2
Low odor	3	A little pleasant	3
Medium odor	4	Neither pleasant nor unpleasant	4
Odor a little strong	5	A little unpleasant	5
Strong odor	6	Medium unpleasant	6
Very strong odor	7	Very unpleasant	7

**Table 3:** Correlations of odor perception with Total Petroleum Hydrocarbons (TPH), Field Capacity and Water repellency.

Sample	Spearman’s Rho Correlations					
	TPH		Field Capacity		Repellency (MED)	
	Intensity	Acceptance	Intensity	Acceptance	Intensity	Acceptance
FLMC-W/O F	0.500	−0.167	0.250	0.317	0.617	0.500
FLMC-WF	0.217	−0.133	0.617	0.467	0.330	0.317
FLHC-W/O F	0.267	0.450	−0.151	−0.176	0.333	0.517
FLHC-WF	0.333	−0.083	0.276	0.042	0.800 *	0.317
GLMC-W/O F	0.360	0.092	0.326	0.025	0.122	0.021
GLMC-WF	0.395	0.151	0.353	0.594	−0.043	−0.153
GLHC-W/O F	0.377	−0.008	−0.105	−0.088	0.201	−0.134
GLHC-WF	0.509	0.286	−0.426	−0.489	0.153	−0.202
ARMC-W/O F	0.619	0.285	0.276	0.460	0.569	0.251
ARMC-WF	0.577	0.577	0.510	0.510	0.544	0.544
ARHC-W/O F	0.467	0.460	−0.159	−0.391	0.250	0.209
ARHC-WF	0.385	0.435	−0.424	−0.256	0.385	0.351

* unique direct correlation with high intensity.

**Table 4:** Evaluation: when they say “it is good to plant”, is it really satisfactory?—Fluvisol.

Sample	Soil Parameters						Odor				Is It Good to Plant?					False Positives	
	Water Repellency				% Reduction in Field Capacity	Toxicity % Mortality	Intensity		Acceptance		Soil Parameters			Odor Perception			
	Severity		Persistence				Value	Classifica-tion	Value	Classifica-tion	REP.	F.C.	TOX.	Intensity	Accept.	TOX.	FERT.
	MED	Classify-cation	WDPT (s)	Classifi-cation													
FLMC-W/O F	2.11	Moderate	209.50	Strong	38.35	0	2.36	Slight–low	2.73	Medium Pleasant–A Little Pleasant	Yes *	No	Yes	Yes	Yes	No	Yes
FLMC-WF	2.81	Severe	>3,600	Extreme	43.54	0	2.56	Slight–low	3.00	A Little Pleasant	Yes *	No	Yes	Yes	Yes	No	Yes
FLHC-W/O F	3.46	Very severe	609.59	Severe	42.73	0	2.51	Slight–low	2.69	Medium Pleasant–A Little Pleasant	Yes *	No	Yes	Yes	Yes	No	Yes
FLHC-WF	3.76	Very severe	>3,600	Extreme	28.30	0	2.58	Slight–low	2.78	Medium Pleasant–A Little Pleasant	Yes *	No	Yes	Yes	Yes	No	Yes

REP. = Repellency, F.C. = Field Capacity, TOX. = Toxicity, FL = Fluvisol, MC = Medium Crude Oil, HC = Heavy Crude Oil, W/O F = Without Fertilizer, WF = With Fertilizer. * At field conditions, these are probably not repellent, Accept. = acceptance, FERT. = Fertility.

**Table 5:** Evaluation: When they say “it is good to plant”, is it really satisfactory?—Gleysol.

Sample	Soil Parameters						Odor				Is It Good to Plant?					False positives	
	Water Repellency				% Reduction in Field Capacity	Toxicity % Mortality	Intensity		Acceptance		Soil Parameters			Odor perception			
	Severity		Persistence				Value	Classifica-tion	Value	Classifica-tion	REP.	F.C.	TOX.	Intensity	Accept.	TOX.	FERT.
	MED	Classify-cation	WDPT (s)	Classifi-cation													
GLMC-W/O F	0.00	Not repellent	12.83	Slight	56.03	0	2.02	Slight–low	2.33	Medium Pleasant–A Little Pleasant	Yes	No	Yes	Yes	Yes	No	N.A.
GLMC-WF	0.00	Not repellent	25.33	Slight	51.35	0	2.02	Slight–low	2.30	Medium Pleasant–A Little Pleasant	Yes	No	Yes	Yes	Yes	No	N.A.
GLHC-W/O F	0.62	Low	89.04	Strong	44.51	0	2.18	Slight–low	2.56	Medium Pleasant–A Little Pleasant	Yes	No	Yes	Yes	Yes	No	N.A.
GLHC-WF	2.00	Moderate	246.76	Strong	39.46	0	2.27	Slight–low	2.60	Medium Pleasant–A Little Pleasant	Yes *	No	Yes	Yes	Yes	No	N.A.

REP. = Repellency, F.C. = Field Capacity, TOX. = Toxicity, GL = Gleysol, MC = Medium Crude Oil, HC = Heavy Crude Oil, W/O F = Without Fertilizer, WF = With Fertilizer. N.A. = Does not apply. In the field these soils retain abundant moisture (>80% Field Capacity). * At field conditions, these are probably not repellent, Accept. = acceptance, FERT. = Fertility.

**Table 6:** Evaluation: when they say “it is good to plant”, is it really satisfactory?—Arenosol.

Sample	Soil Parameters						Odor				Is It Good to Plant?					False Positives	
	Water Repellency				% Reduction in Field Capacity	Toxicity % Mortality	Intensity		Acceptance		Soil Parameters			Odor Perception			
	Severity		Persistence				Value	Classifica-tion	Value	Classification	REP.	F.C.	TOX.	Intensity	Accept.	TOX.	FERT.
	MED	Classify-cation	WDPT (s)	Classifi-cation													
ARMC-W/O F	5.34	Very severe	>3600	Extreme	48.93	0	3.09	Low–medium	3.27	A Little Pleasant– Neither Pleasant Nor Unpleasant	No	No	Yes	No	Yes	No	Yes
ARMC-WF	5.12	Very severe	>3600	Extreme	58.82	0	2.49	Slight–low	3.02	A Little Pleasant– Neither Pleasant Nor Unpleasant	No	No	Yes	No	Yes	No	Yes
ARHC-W/O F	5.46	Very severe	>3600	Extreme	23.23	0	3.24	Low–medium	3.62	A Little Pleasant– Neither Pleasant Nor Unpleasant	No	No	Yes	No	Yes	No	Yes
ARHC-WF	5.34	Very severe	>3600	Extreme	19.90	0	3.73	Low–medium	3.93	A Little Pleasant– Neither Pleasant Nor Unpleasant	No	No	Yes	No	Yes	No	Yes

## Supplementary Materials

The following are available online at https://www.mdpi.com/1660-4601/17/9/3213/s1, Figure S1: Experimental design, Figure S2: Form used in the olfactory perception test, Figure S3: Acceptance form for participation in an investigation, Figure S4: Panelist data sheet, Figure S5: TPH degradation in contaminated soils with medium crude oil without fertilizer, Figure S6: TPH degradation in contaminated soils with medium crude oil with fertilizer, Figure S7: TPH degradation in contaminated soils with heavy crude oil without fertilizer, Figure S8: TPH degradation in contaminated soils with heavy crude oil with fertilizer, Figure S9: Field capacity in contaminated soils with medium and heavy crude oil, Table S1: Characterization of uncontaminated soils, Table S2: Initial fertility parameters in contaminated soils, Table S3: Classification of the severity of water repellency evaluated by the MED method and expressed as molarity, proposed by King (1981), Table S4: Classification of the WDPT proposed by Dekker and Jungerius, Table S5: Repellency (MED y WDTP) in contaminated soils treated by bioremediation and natural attenuation for 18 months, Table S6: Weight loss of organisms in acute toxicity tests by direct contact, Text: Project Ethics Details.
