# Supplementary material for: Soil Odor as An Extra-Official Criterion for Qualifying Remediation Projects of Crude Oil-Contaminated Soil
Source: Int J Environ Res Public Health. 2020 May 5;17(9):3213. doi: 10.3390/ijerph17093213 (PMC7249263; doi:10.3390/ijerph17093213)
Supplement: Supplementary file 1 [file ijerph-17-03213-s001.pdf]

Supplementary Material

Soil odor as an extra-oficial criterion for qualifying remediation projects of crude oil-contaminated soil.

Saúl López-Aguilar, Randy H. Adams, Verónica I. Domínguez-Rodríguez, José A. Gaspar-Génico, Joel Zavala-Cruz, Edith Hernández-Natarén

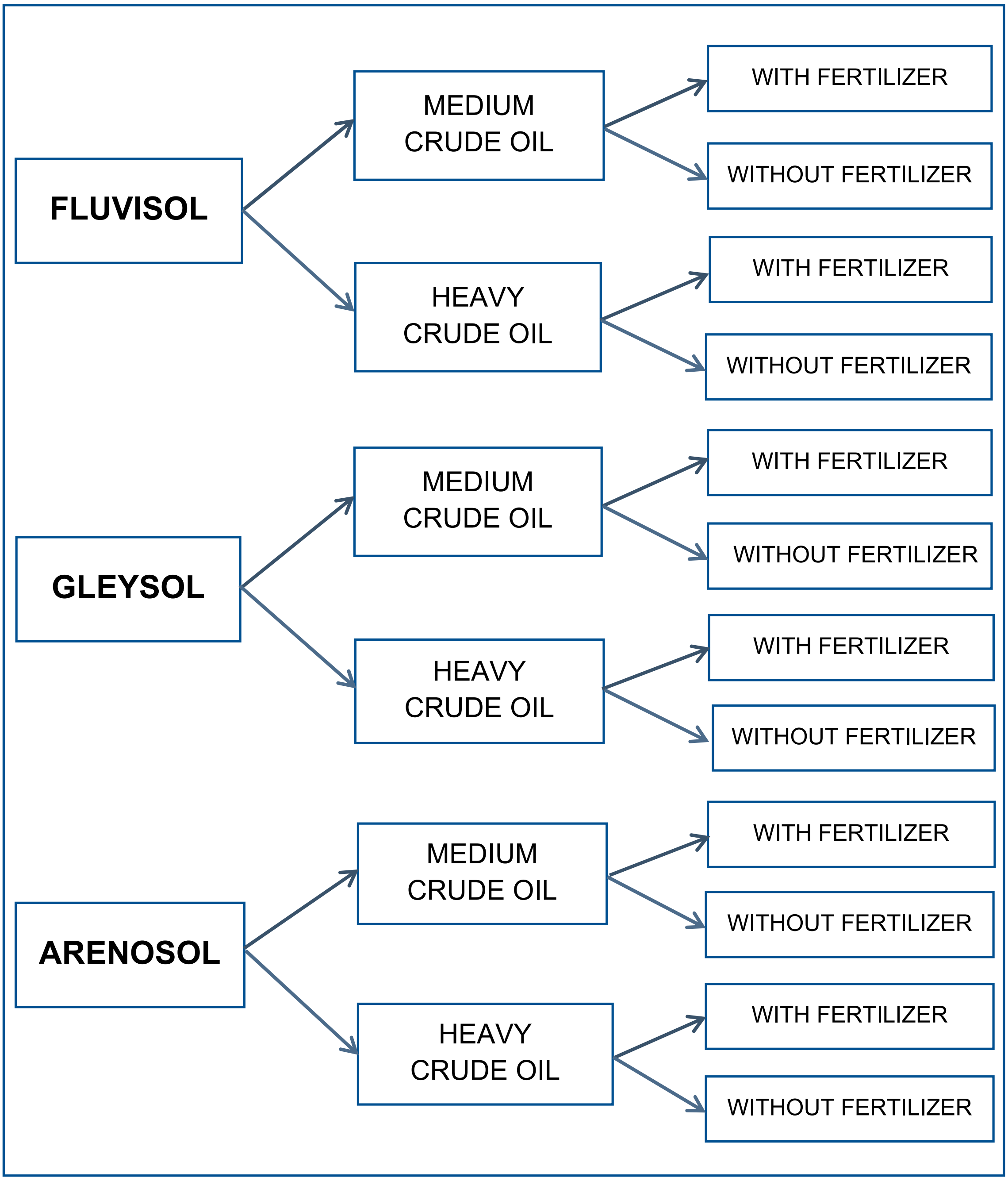

Figure S1. Experimental design.

Project Ethics Details.

The research protocol was evaluated and approved by the Divisional Research Committee of the Biological Sciences Academic Division of the Univerisidad Juárez Autónoma de Tabasco (Folio No. 395-2017) after considering protection to human health, dignity and scientific rigor.

Although this study did not involve clinical trials, it did involve human panelist. As such, it was felt to be important to consider some factors commonly used in clinical trials. Panelist received information previous to the test with respect to the test objectives, possible risks, test procedure, as well as the complete freedom to abandon the test at any moment if they choose to. Also, their understanding and acceptance was confirmed by a signed consent form (Figure S3, Supplementary Material). Likewise, some personal data was solicited (Figure S4, Supplementary Material). Panelist participated voluntarily, without any coercion or monetary remuneration, nor reprisal for failing to participate or withdrawing themselves from the test.

It is worth mentioning that before approval of the study, a risk evaluation was made for the panelists' exposure. The Recommended Exposure Level (REL) approved by the National Institute of Occupational Safety and Health – NIOSH [45] for a similar mixture of hydrocarbons (Kerosene), is 100 mg m^{-3}. If one considers that the average person inhales roughly 20 m^3 of air a day, and that a works shift is eight hours a day, the corresponding REL in terms of milligrams of hydrocarbon inhaled per day per person = (20 m^3 air/24 h) (8 hours) (100 mg m^{-3}) = 667 mg d^{-1} person^{-1} (over one-half gram per day).

The concentration and exposure time of the panelist was much less than this. The exposure to each sample was approximately 5 – 10s each, for a lot of 36 samples = 360s maximum, or less than six minutes over a test of 30-40 minutes. The vapor concentration was estimated to be less than 1/10th that of an occupational exposure based on organoleptic observations. None-the-less, if one considers a maximum concentration as high as an occupational exposure (100 mg m^{-3}), the daily exposure for the sum of 36 samples, in terms of milligrams of hydrocarbons inhaled per person per day = (20 m^3 air/24 h) (hr/60 min.) (6 min.)(100 mg m^{-3}) = 8.3 mg d^{-1}person^{-1}, or less than 80 times the recommended exposure level.

After reviewing these data, the Divisional Research Committee and the Environmental Engineering Academic Research Group at the university considered that the risks to panelists were negligible and that the protocols were adequate to protect human health and dignity. It should also be noted that no panelist complained of symptoms typical of over-exposure to hydrocarbons by inhalation (such as nasal congestion, difficulty breathing, headache or nausea), and no panelist asked to withdraw from the test.

**PRUEBA DE PERCEPCION OLFATIVA**											
**No. de Muestra:**					**Fecha:**			**Hora:**			
**Nombre:**											
**Instrucciones**: Lea con atención cada pregunta y marque con una **X** el cuadro que consideres contiene la respuesta correcta											
**¿Huele a tierra normal?**		**¿Huele a tierra buena?**		**Intensidad de olor ¿Huele a petróleo crudo?**			**Nivel de aceptación del olor ¿Es agradable o desagradable?**			**¿Sirve para sembrar? (está bien)**	
Si	No	Si	No	Sin olor	1		Muy agradable	1		Si	No
				Olor ligero (apenas perceptible)	2		Medio agradable	2			
				Olor bajo	3		Un poco agradable	3			
				Olor mediano	4		Ni agradable ni desagradable	4			
				Olor un poco fuerte	5		Un poco desagradable	5			
				Olor fuerte	6		Medio desagradable	6			
				Olor muy fuerte	7		Muy desagradable	7			

a) – Original form

**OLFACTORY PERCEPTION TEST**											
**Sample No.:**					**Date:**			**Time:**			
**Nombre:**											
**Instructions:** Read each question carefully and mark with an X the box you consider contains the correct answer											
**Does it smell like normal earth?**		**Does it smell good ground?**		**Odor intensity** Does it smell like crude oil?			**Acceptance level for odor** Is it pleasant or unpleasant?			**Does it serve to sow? (it's okay)**	
Yes	No	Yes	No	Without odor	1		Very pleasant	1		Yes	No
				Slight odor (barely perceptible)	2		Medium pleasant	2			
				Low odor	3		A little pleasant	3			
				Medium odor	4		Neither pleasant nor unpleasant	4			
				Odor a little strong	5		A little unpleasant	5			
				Strong odor	6		Medium unpleasant	6			
				Very strong odor	7		Very unpleasant	7			

b) - Translated form

Figure S2. a), b) Format used in the olfactory perception test.

FORMATO DE ACEPTACION DE PARTICIPACION EN UNA INVESTIGACION [1]

Lugar: ____________________________________Fecha: ______________________

A QUIEN CORRESPONDA

Yo ____ declaro libre y voluntariamente que acepto participar en el estudio "**Percepción olfativa como criterio para evaluar la efectividad de la restauración de suelos contaminados con petróleo crudo"** que se realizará en la Institución cuyos objetivos consisten en evaluar la efectividad de las pruebas de percepción olfativa como criterio para la valoración de las obras de restauración de suelos contaminados con petróleo crudo mediano y pesado.

Estoy consciente de que los procedimientos, pruebas y tratamientos, para lograr los objetivos mencionados consistirán en hacer una prueba de olor controlada a las muestras presentadas y que los riesgos a mi persona tienen pocas posibilidades.

Entiendo que del presente estudio se derivarán los siguientes beneficios:

- Contribuir en la generación de conocimiento mediante una publicación científica que realizará nuestra institución.
- Demostrar que la percepción olfativa es un criterio que se puede emplear en la restauración de suelos contaminados con petróleo crudo.

Es de mi conocimiento que seré libre de retirarme de la presente investigación en el momento que así yo lo desee. También que puedo solicitar información adicional acerca de los riesgos y beneficios de mi participación en este estudio. En caso de que decidiera retirarme, la atención que como trabajador (o estudiante) recibo en esta institución no se verá afectada.

Nombre ____________________________________ Firma ________________________

Dirección ____

Fecha ____

Testigo ____

Dirección ____

Testigo ____

Dirección ____

3a) – Original form

CONSENT FORM FOR PARTICIPATION IN RESEARCH STUDY [1]

Place: ______________________________________Date: _______________________

TO WHOM IT MAY CONCERN

I, ____ freely and voluntarily declare that I accept to participate in the study titled "**Olfactory perception as a criterion for evaluating the effectiveness of the restoration of crude oil-contaminated soils"** que to be conducted in the Institution, and whose objectives consist of assessing the effectiveness of olfactory perception tests as a criterion for the evaluation of projects aimed at the restoration of soils contaminated with medium and heavy crude oil.

I understand the procedures, tests and treatments used to achieve the above mentioned objectives, and that this consists in conducting a controlled odor test on those samples that are presented and also the risks to my person has a low probability.

I understand that from the present study the following benefits may be derived:

- Contribution to the generation of knowledge through a scientific publication that our institution will carry-out.
- Demostration thar olfactory perception is a criterion that may be employed in the resotration of crude oil-contaminated soils.

It is my understanding that I will be free to remove myself from the present research at any moment that I may desire to do so. Also, that I may solicit additional information about the risks and benefits of my participation in the study. In case I decide to remove myself, the treatment that I receive as a worker (or student) in the institution will not be affected.

Name ____________________________________ Signature _______________________

Address ____

Date ____

Witness ____

Address ____

Witness ____

Address ____

3b) - Translated form

Figure S3. Acceptance form for participation in an investigation.

PROYECTO

"Percepción olfativa como criterio para evaluar la efectividad de la restauración de suelos contaminados con petróleo crudo"

HOJA DE DATOS DE PANELISTAS **Fecha____________________**

Nombre:		Edad:
Sexo: M ☐ F ☐	Ocupación:	
Domicilio:		
Lugar de Origen:	Zona Urbana ☐ Zona Rural ☐	
¿Conoces el objetivo de la investigación?		Si ☐ NO ☐
¿Estás convencido de querer participar?		Si ☐ NO ☐
¿Eres fumador?		Si ☐ NO ☐
¿Consumes café con regularidad?		Si ☐ NO ☐
¿Eres alérgico a alguna sustancia?		Si ☐ NO ☐
¿Cómo consideradas tu estado de salud?		Bueno ☐ Malo ☐
¿Has visto algún derrame de petróleo?		Si ☐ NO ☐
¿Has trabajado en la industria petrolera?		Si ☐ NO ☐

4a) - Original form

PROJECT

"Olfactory perception as a criterion for evaluating the effectiveness of the restoration of crude oil-contaminated soils"

PANELIST DATA SHEET **Date:**______________________________

Nomber:		Age:	
Sex: M ☐ F ☐	Occupation:		
Address:			
Birthplace:	Urban Zone ☐ Rural Zone ☐		
Do you know the purpose of the investigation?		Yes ☐	NO ☐
Are you convinced of wanting to participate?		Yes ☐	NO ☐
Are you a smoker?		Yes ☐	NO ☐
Do you consume coffee regularly?		Yes ☐	NO ☐
Are you allergic to any substance?		Yes ☐	NO ☐
How do you consider your state of health?		Good ☐	Bad ☐
Have you seen any oil spills?		Yes ☐	NO ☐
Have you worked in the oil industry?		Yes ☐	NO ☐

4b) Translated data sheet

Figure S4. Panelist data sheet

Table S1. Characterization of uncontaminated soils [2]

Variable	Fluvisol	Arenosol	Gleysol
Coordinates	18° 06′ 04′′N, 93° 52′ 59′′W	18° 12′ 47′′N, 94° 00′ 42′′W	18° 06′ 28′′N, 93° 52′ 57′′W
Real Density (g cm^{-3})	2.4	2.5	2.2
Apparent Density (g cm^{-3})	1.1	1.4	1.1
pH	7.0	6.7	7.1
Porosity (%)	54	45.6	49.5
Field Capacity (%)	37	19.5	37
Electric Conductivity (dS m^{-1})	0.01	0.31	0.01
Organic material (%)	2.3	1.46	4.9
Texture	Clayey	Sandy	Clayey
Sand (%)	6.8	87.2	25.8
Silt (%)	34.3	3.7	21.3
Clay (%)	58.8	9.0	52.8
Cation Exchange Capacity (cmol(+) kg^{-1})	21.4	3.1	35.7
N (cmol kg^{-1})	0.8	0.056	0.2
P Olsen (mg kg^{-1})	23.38	2.75	11.69
Ca^{++} (cmol kg^{-1})	19	1.39	25.53
Mg^{++} (cmol kg^{-1})	1.3	0.36	2.06
Na^{+} (cmol kg^{-1})	0.2	0.12	1.34
K^{+} (cmol kg^{-1})	0.2	0.05	0.53
WDPT (s)	<5	<5	<5
MED (M)	0	0	0

Table S2. Initial fertility parameters in contaminated soils

SOIL TYPE	Field Capacity (%)			Repellency (MED-10) (molar)			Repellency (WDPT) (s)		
	Soil Clean	Contaminated Soil		Soil Clean	Contaminated Soil		Soil Clean	Contaminated Soil	
		W/O F	WF		W/O F	WF		W/O F	WF
FLMC	37	35.13	34.62	0	5.28	5.59	<5	>3,600	>3,600
FLHC	37	26.64	26.27	0	6.22	6.23	<5	>3,600	>3,600
GLMC	19.5	18.21	18.21	0	11	10.58	<5	>3,600	>3,600
GLHC	19.5	23.09	23.09	0	6.33	6.22	<5	>3,600	>3,600
ARMC	37	37.21	35.73	0	3.78	3.24	<5	137.78	245.49
ARHC	37	27.9	30.14	0	5.24	5.37	<5	1,944.81	>3,600

FL=Fluvisol; GL=Gleysol; AR=Arenosol; MC= Medium Crude Oil; HC=Heavy Crude Oil; W/O F=Without Fertilizer; WF=With Fertilizer

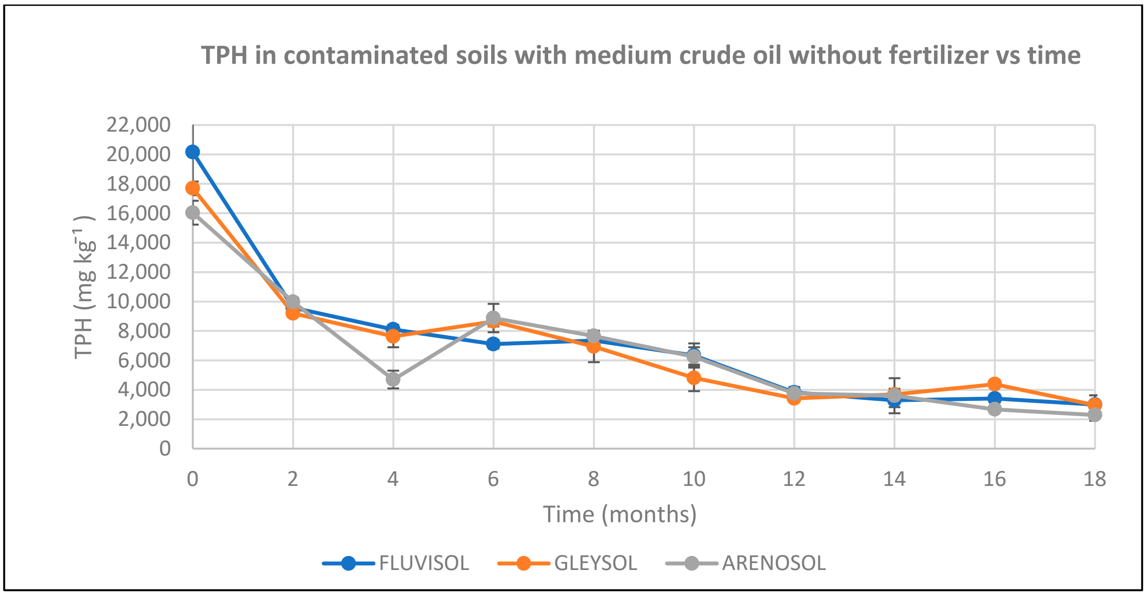

Figure S5. TPH degradation in contaminated soils with medium crude oil without fertilizer.

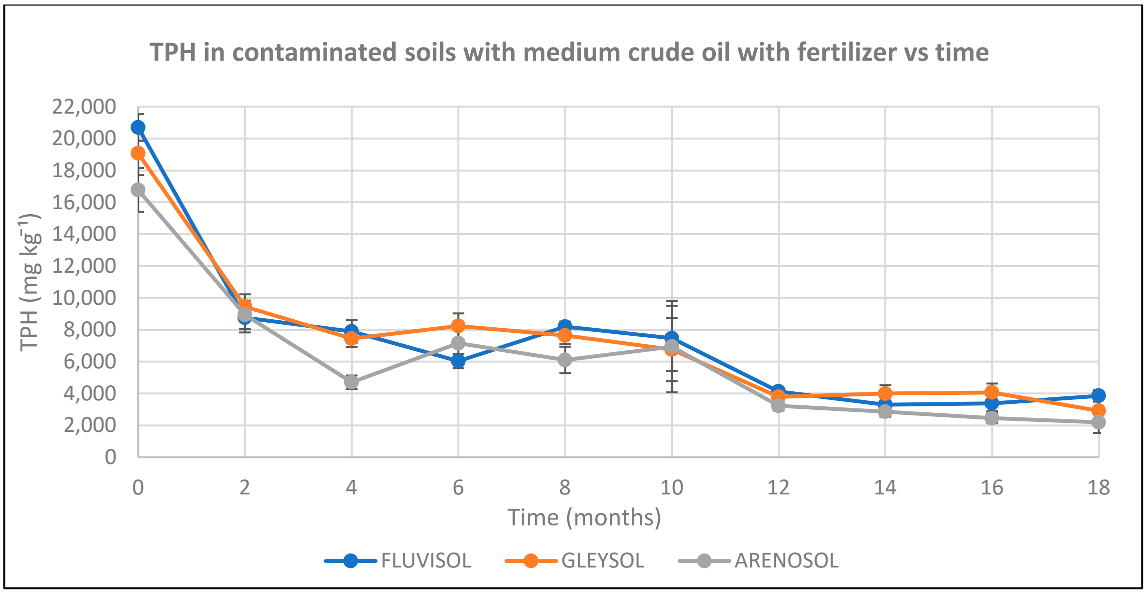

Figure S6. TPH degradation in contaminated soils with medium crude oil with fertilizer.

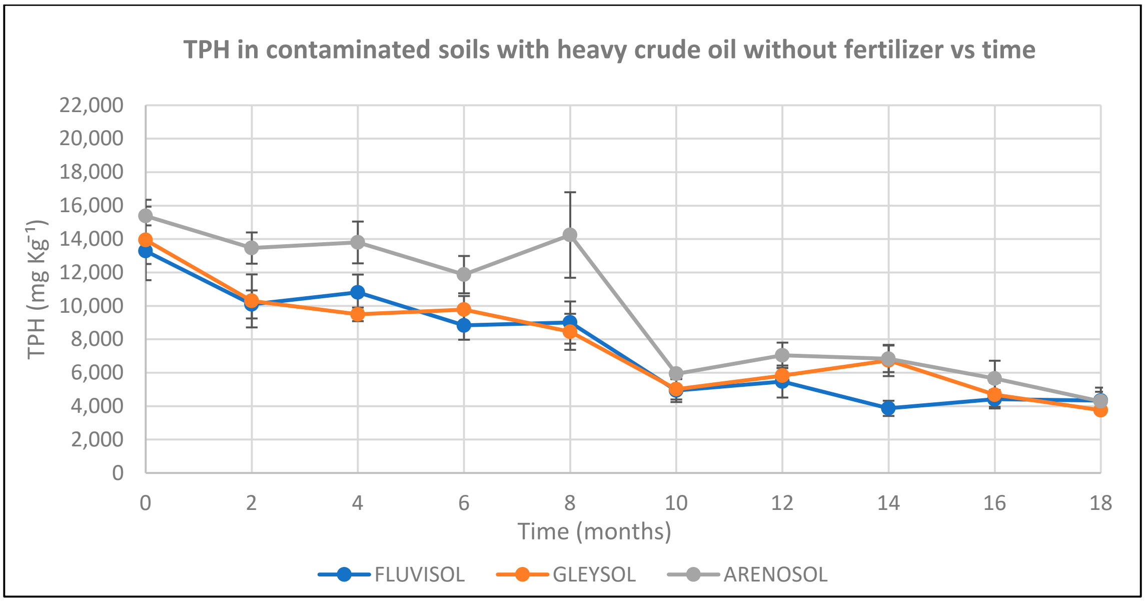

Figure S7. TPH degradation in contaminated soils with heavy crude oil without fertilizer.

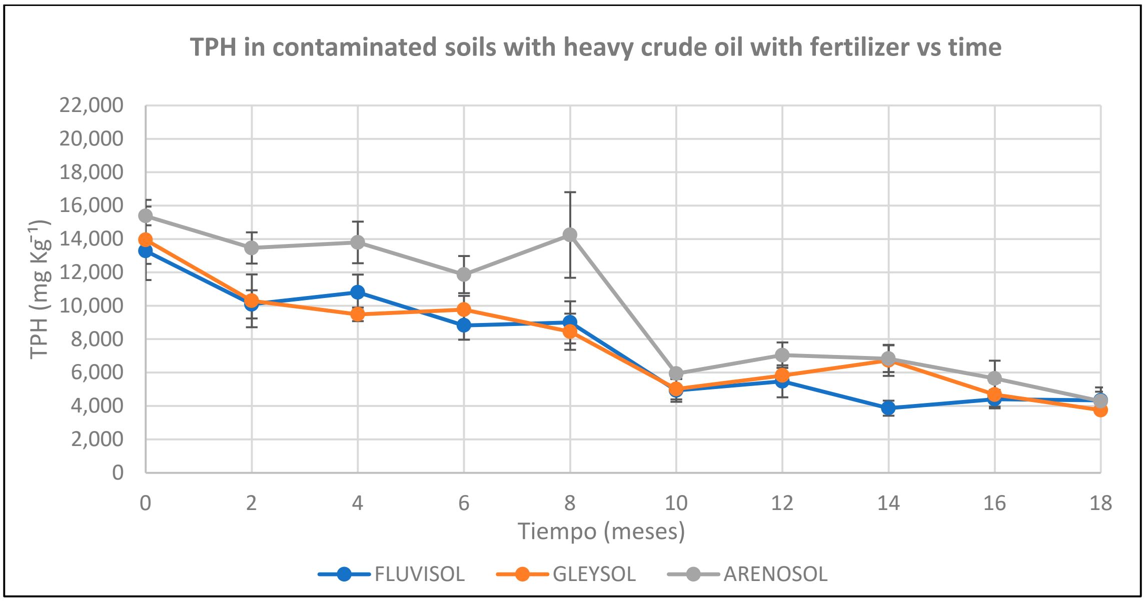

Figure S8. TPH degradation in contaminated soils with heavy crude oil with fertilizer.

Table S3. Classification of the severity of water repellency evaluated by the MED method and expressed as molarity, proposed by King (1981). Source: Jaramillo [3].

Severity class	MED (M) value range
Not repellent	0
Low repellency	0.2 - 1.0
Moderate repellency	1.2 - 2.2
Severe repellency	2.4 – 3.0
Very severe repellency	>3.2

Table S4. Classification of the WDPT proposed by Dekker & Jungerius [4]

Persistence class	Persistence level of water repellency	WDTP (s) values
0	Not repellent	<5
1	Light	5 - 60
2	Strong	60 - 600
3	Severe	600 - 3600
4	Extreme	>3600

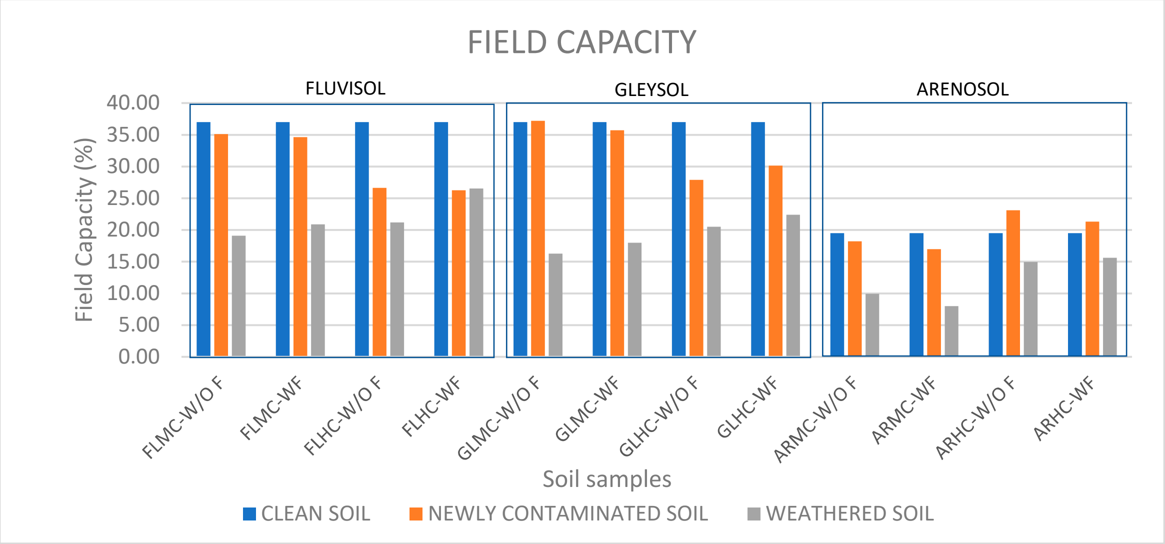

Figure S9. Field capacity in contaminated soils with medium and heavy crude oil

FL=Fluvisol; GL=Gleysol; AR=Arenosol; MC= Medium Crude Oil; HC=Heavy Crude Oil; W/O F=Without Fertilizer; WF=With Fertilizer

Table S5. Repellency (MED y WDTP) [5] in contaminated soils treated by bioremediation and natural attenuation for 18 months

Sample	REPELLENCY (MED-10) (molarity)		REPELLENCY **WDTP (s)**	
	MED Treated soil	Severity Classification	WDTP Treated soil	Persistence Classification
FLMC-W/O F	2.11	Moderate	209.50	Strong
FLMC-WF	2.81	Severe	>3,600	Extreme
FLHC-W/O F	3.46	Very severe	609.59	Severe
FLHC-WF	3.76	Very severe	>3600	Extreme
GLMC-W/O F	0.00	Not Repellent	12,83	Light
GLMC-WF	0.00	Not Repellent	25.33	Light
GLHC-W/O F	0.62	Low	89.04	Strong
GLHC-WF	2.00	Moderate	246.76	Strong
ARMC-W/O F	5.34	Very Severe	>3,600	Extreme
ARMC-WF	5.12	Very Severe	>3,600	Extreme
ARHC-W/O F	5.46	Very Severe	>3,600	Extreme
ARHC-WF	5.34	Very Severe	>3,600	Extreme

FL=Fluvisol; GL=Gleysol; AR=Arenosol; MC= Medium Crude Oil; HC=Heavy Crude Oil; W/O F=Without Fertilizer; WF=With Fertilizer

Table S6. Weight loss of organisms in acute toxicity tests by direct contact.

SAMPLE	October 2018		December-2018	
	Weight loss		Weight loss	
	Sample	Witness	Sample	Witness
FLMC-W/O F	14.59%	5.81%	15.88%	15.86%
FLMC-WF	12.17%	5.81%	13.05%	15.86%
FLHC-W/O F	17.28%	17.49%	20.35%	17.42%
FLHC-WF	10.63%	17.49%	18.91%	17.42%
GLMC-W/O F	11.66%	5.81%	14.86%	12.10%
GLMC-WF	8.15%	5.81%	8.91%	12.10%
GLHC-W/O F	12.27%	17.49%	19.70%	18.69%
GLHC-WF	12.44%	17.49%	19.33%	18.69%
ARMC-W/O F	21.30%	-2.67%	24.80%	12.02%
ARMC-WF	17.80%	-2.67%	18.15%	12.02%
ARHC-W/O F	27.31%	-2.67%	18.78%	6.85%
ARHC-WF	20.36%	-2.67%	16.32%	6.85%

FL=Fluvisol; GL=Gleysol; AR=Arenosol; MC= Medium Crude Oil; HC=Heavy Crude Oil; W/O F=Without Fertilizer; WF=With Fertilizer

References for Supplementary Material:

1. Mendez-Ramirez, I., et al., *El protocolo de Investigación: Lineamientos para su elaboración y análisis (The Research Protocol: Guidelines for its elaboration and analysis)*. 2a ed. 2011, México: Trillas. 210 p.

2. Ávila Acosta, C.R., *Efectos de la intemperización en las propiedades fisicoquímicas del suelo contaminado con petróleo crudo. (Tesis de Maestría). [Effects of weathering on the physiochemical properties of crude oil-contaminated soil. (Master's Thesis)]*. 2014, Universidad Juárez Autónoma de Tabasco, División Académica de Ciencias Biológicas: Villahermosa, Tabasco, México.

3. Jaramillo, J., Repelencia al agua en suelos: una síntesis (Water repellency in soils: a synthesis). *Rev. Acad. Colomb. Cienc*, 2006. **30**(115): p. 215-232.

4. Dekker, L.W. and P.D. Jungerius, Water repellency in the dunes with special reference to the Netherlands. *Catena, Supplement*, 1990(18): p. 173-183.

5. Adams, R.H., F.J. Guzmán-Osorio, and J. Zavala-Cruz, Water repellency in oil contaminated sandy and clayey soils. *International Journal of Environmental Science and Technology*, 2008a. **5**(4): p. 445-454.
